# Sciatica*Read before the Illinois State Veterinary Medical Association.

**Published:** 1892-04

**Authors:** John Scott


### SCIATICA.*

BY JOHN SCOTT.

During the summer of 1890, I was called upon to treat the following case which I diagnosed as sciatica, and as I have only seen two such cases during a practice of six years, both of which occurred under conditions almost identical, and both in horses kept for racing purposes, I judge that such cases are somewhat rare in our patients. I am forced to this conclusion, not because I have only been fortunate enough to see two cases, but from the fact that in all the works on veterinary science that I have had an opportunity of reading, I fail to find such a disease even mentioned, and in our Veterinary Journals the only place I see such a disease referred to is in the March, 1889 number of the *Review*, where a case is recorded; but that is an extract from a foreign journal. For those reasons I call your attention to it to-day, and hope it may prove as interesting to you as it did to me.

I was called to the case on Wednesday, July 22, 1890, during our summer race-meeting, and on reaching the driving park, found that the animal affected was a seven-year-old pacing gelding; record: 2.23¼.

I asked for and obtained the following history of the case from his owner. He had been started in his first race of the season, at Bloomington, the first week in July; next week was in a hard contested race at Springfield, after which he went a trifle lame in near hind leg; the next week he was in Decatur, but was not started; from Decatur was shipped to Peoria, arriving here on Sunday, July 19th; on Monday, Tuesday and Wednesday mornings he was jogged for a few miles on the track, but being still slightly lame, his owner concluded to ship him home until fully recovered ; but when they started to lead him to the shipping platform, they only got about three hundred yards from the stall, when he was taken so suddenly and violently lame, and seemed to be in such agony, that it was almost impossible to get him back to the stall, where I saw him about half an hour later, presenting the following symptoms:

His general appearance showed at a glance that he was suffering intense pain of a nervous character, there being nervous twichings, slight clonic spasms of the muscles, holding the near hind leg

*Read before the Illinois State Veterinary Medical Association.

clear of the floor, but continually moving it in a convulsive, jerky, nervous manner, nostrils dilated, breathing very much accelerated, pulse 85 and weak, temperature 103, body covered with perspiration.

A close and careful examination of the foot and leg revealed nothing wrong, but on manipulating the hip and thigh, the symptoms were very much aggravated; the poor animal groaning from pain, on pressure being brought to bear on those parts, and clonic spasm of the muscles being much more severe. I gave Potassium Bromide 1 drachm, and also Morph. Sulph. four grains hypodermically; injected a five per cent. solution of Cocaine into muscles of hip and thigh, and ordered hot woollen blankets applied to the to the hip. On my return in about two hours I found him in about the same condition, suffering intensely, sweating profusely, but body cold. To satisfy the owner I passed the catheter, and drew off a small quantity of natural-looking urine. I then gave him Potassium Bromide 1 drachm, Chloral Hydrate one drachm and Gelsemium Fl. Ex. 1 drachm; in an hour repeated the Bromide and Gelsemium, and after that every two hours; kept hot blankets to the hip, and used an anodyne liniment: at 10 P. M. he was feeling easier, so I left him in charge of an attendant, with instructions to give two doses of Bromide and Gelsemium, during the night.

I saw him next morning about five o'clock, when he was looking and feeling better; he had turned round in his stall during the night, being the first time he had changed his position, but would bear no weight on the affected limb. I continued giving the Bromide and Gelsemium every four or five hours, gradually decreasing the dose as the nervous symptoms disappeared, and on the fifth day discontinued them entirely, and prescribed tonics, as his appetite was very poor.

During this time I had been treating the hip and thigh with hot blankets and anodyne liniments, but as the gluteal muscles began to show signs of becoming atrophied and the pain still being severe, I applied a blister to the parts The third day after applying it we led him out of the stall for the first time, but he would scarcely bear any weight on the affected limb, merely touching the toe to the ground; but as the owner was anxious to get away, we concluded to try and move him to my hospital, which we did the following night, where he lay down for the first time; he lay down pretty regular after this, but had to be helped up for several days. I continued giving him tonics for about three weeks before he regained his appetite, and by this time he wasn't much more than a shadow, but was able to walk out for a little exercise, and from this time on he improved quite rapidly.

The gluteal muscles became very much atrophied, but this I finally overcame by repeated blistering. In about six weeks from the time he was admitted to the hospital I put him in harness for the first time and drove him a few blocks; after this gave him regular exercise, and in sixty days from the date of admission I sent him home, all right in every way except that the gluteal muscles were still slightly atrophied; but I had an opportunity of seeing him again in about six weeks, and at that time they were entirely filled out and had regained their natural appearance.

The other case I saw was during our race-meeting in July, 1891, and occurred in a seven-year trotting gelding, record 2.22¾. He started in a race on Thursday, winning two heats, when darkness came, and the race was postponed; the next day he came out all right, but in scoring down for the first heat he went suddenly lame in near hind leg—so lame that it was difficult to get him to his stall. He presented the same symptoms in general that the other horse did, but not nearly so severe; I treated him the same as my first case, and in about a week he was sufficiently recovered to be shipped home.

